# Rare Adventitial Venous Cyst Mimicking Deep Vein Thrombosis: A Diagnostic Pitfall—A Systematic Review of Diagnostic Challenges, Imaging Features, and Optimal Treatment Strategies

**DOI:** 10.3390/jcm15093314

**Published:** 2026-04-27

**Authors:** Maciej Nowacki, Adam Nowacki, Marcin Tukiendorf, Ireneusz Wiernicki, Ryan Stolze

**Affiliations:** 1 Department of Vascular Surgery and Angiology, Pomeranian Medical University in Szczecin, 70-204 Szczecin, Poland; itskorn@gmail.com (A.N.); marcintuki@gmail.com (M.T.); irekwie@wp.pl (I.W.); 2 Department of Anesthesiology and Intensive Care, Pomeranian Medical University in Szczecin, 70-204 Szczecin, Poland; ryan.stolze@icloud.com

**Keywords:** adventitial cystic disease, venous adventitial cystic disease (VACD), venous cyst, deep vein thrombosis (DVT) mimicking, diagnostic mimics, limb edema without deep vein thrombosis (DVT), CT differentiation of the DVT, MRI differentiation of the DVT, misdiagnosis, unilateral leg swelling

## Abstract

Adventitial cystic disease (ACD) is a rare vascular condition, representing approximately 0.1% of all vascular diseases, with about 325 cases reported in the literature since its first description in 1947, including 72 venous cases. This study aims to highlight the diagnostic and therapeutic challenges of venous ACD, which is frequently misdiagnosed as deep vein thrombosis (DVT), femoral varices, aneurysms, venous tumors, or lymphadenopathy due to its rarity. Clinical, imaging, and treatment data from reported cases of venous adventitial cystic disease (VACD) were reviewed. The disease most commonly involved the common femoral vein (56%) and external iliac vein (24%), with less frequent involvement of the saphenous and popliteal veins (7%). Symptoms commonly mimic deep vein thrombosis (DVT), with unilateral swelling resulting from progressive cyst enlargement and subsequent venous luminal stenosis. Doppler ultrasound typically shows a hypoechoic lesion in the venous wall with preserved flow and normal D-dimer levels, while CT angiography and MRI confirm an adventitial cyst occupying ≥ 90% of the lumen without thrombus. Surgical intervention, particularly transadventitial cyst evacuation with excision, is the preferred method of treatment due to lower recurrence rates (7–20%) as compared to cyst aspiration and drainage with higher recurrence (83.3%). These findings highlight the need for heightened clinical suspicion and advanced imaging to optimize the diagnosis and management of venous ACD and reduce misdiagnosis and recurrence. Further studies are needed to optimize diagnostic protocols and treatment strategies, but the limited number of cases hampers meaningful scientific research.

## 1. Introduction

Adventitial cystic disease (ACD) was first described in 1947 involving the external iliac artery [1]. It is a rare vascular condition, accounting for approximately 0.1% of all vascular diseases [2]. To date, approximately 325 cases of adventitial cystic disease (ACD) have been reported, predominantly involving the arterial wall, with only 72 venous cases described since 1963 [3] (Table 1). The condition typically affects men in their fourth or fifth decade of life [4,5]. ACD is characterized by the formation of mucinous cysts within the adventitial layer of arterial or venous walls. It may lead to various signs and symptoms depending on the location and degree of luminal obstruction, including intermittent claudication or rest pain when affecting the popliteal artery (most commonly), as well as limb swelling when localized in veins (85%). Less common manifestations include pain (15%), a palpable mass (28%), varicosities, or paresthesia (5–7%) [3].

The male-to-female ratio in venous adventitial cystic disease is approximately 1.3–1.6:1, compared with a ratio of 5–15:1 in arterial adventitial cystic disease [1,6]. The most common site of venous adventitial cysts is the wall of the common femoral vein (56%), followed by the external iliac vein (24%), with less frequent involvement of the saphenous and popliteal veins (7%) [1] (Table 2). Adventitial cystic disease (ACD) remains a rare and therapeutically challenging condition due to uncertainty regarding its etiology. The rarity of this disease often leads to misdiagnosis or delayed diagnosis, as it may mimic deep vein thrombosis (DVT) [7], femoral varices, femoral aneurysms, venous tumors, or lymphadenopathy [3,6], thereby underscoring the need for a high index of clinical suspicion. The cyst contains mucin, possibly related to overexpression of mucin-associated genes such as MUC1, which is also linked to multiple cancers [8]. Although no evidence of a genetic predisposition to the disease has been reported, a high degree of suspicion may be warranted when family members present with similar symptoms. However, the lack of data due to the limited number of cases does not allow us to confirm this. One case of coincidental arterial and venous adventitial cystic disease (ACD) has been described in a 52-year-old man with ACD in the popliteal artery and a 49-year-old woman with ACD in the popliteal vein (first-degree relatives, a brother and sister) [9]. Overall, only two cases of coexistence (arterial and venous) have been reported. 

The aim of this study is to present a rare case of venous adventitial cystic disease (VACD) in the common femoral vein of a 33-year-old male, to review current literature on its diagnostic challenges and treatment strategies, and to propose a clinical algorithm for differentiating VACD from DVT.

## 2. Case Presentation

Clinical symptoms often resemble those of deep vein thrombosis (DVT), most commonly presenting with unilateral limb swelling. Progressive enlargement of venous adventitial cystic disease results in luminal stenosis and subsequent clinical symptoms. Doppler ultrasound typically demonstrates a hypoechoic lesion causing venous luminal narrowing, occasionally associated with diffuse enlargement of the affected vein [3].

A 33-year-old male soldier was referred to our hospital with nonspecific lower limb symptoms. His medical history was notable for blunt trauma to the groin sustained during a bicycle accident in childhood. As an adult, he reported a persistent sensation of leg heaviness and altered limb perception, which he described as a “wooden leg.”

His symptoms gradually worsened over a 2-year period, with progressively increasing, non-painful swelling that was most pronounced after physical exertion. Eventually, he developed persistent swelling of the entire limb extending from the groin.

The patient was initially referred by his general practitioner to an orthopedic surgeon, who performed a physical examination and Doppler ultrasound before referring him to the Vascular Surgery Clinic with suspected deep vein thrombosis.

On physical examination, the entire lower limb was edematous from the groin distally, without erythema or increased local temperature.

Doppler ultrasound demonstrated a 25 mm long hypoechoic mass situated within the common femoral vein, causing critical luminal stenosis with preserved blood flow in the femoral vein and normal D-dimer levels D-dimer (0.38 mg/L FEU, reference < 0.5).

Due to the nonspecific clinical presentation, the presence of a solitary atypical structure not characteristic of venous thrombosis, and its limitation to the common femoral vein, further detailed diagnostic evaluation was undertaken.

**Figure 1:**
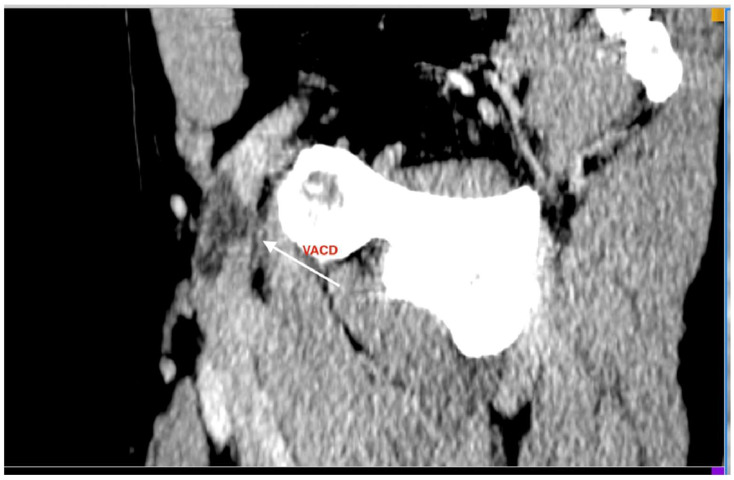
Axial contrast-enhanced computed tomography angiography demonstrating venous adventitial cystic disease; the arrow indicates the cystic lesion.

**Figure 2:**
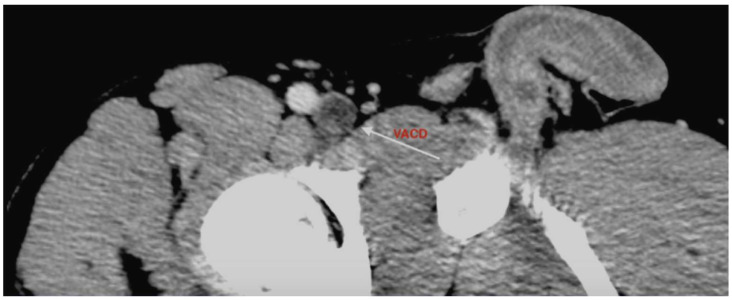
Cross-sectional CT angiography image demonstrating venous adventitial cystic disease; the arrow indicates the cystic lesion. The structure fills almost the entire lumen.

**Figure 3:**
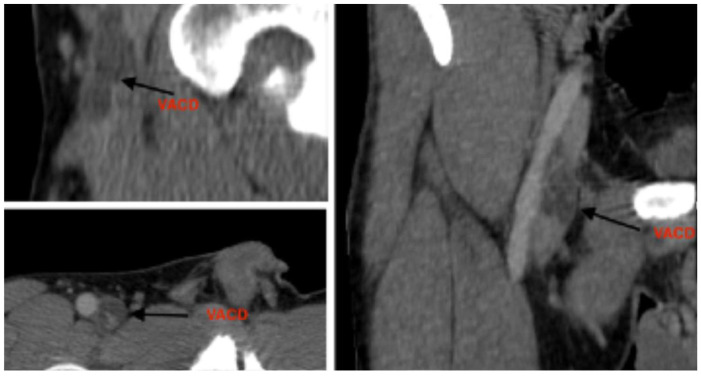
CT angiography image demonstrating venous adventitial cystic disease; the arrow indicates the cystic irregular lesion. The structure fills almost the entire lumen.

**Figure 4:**
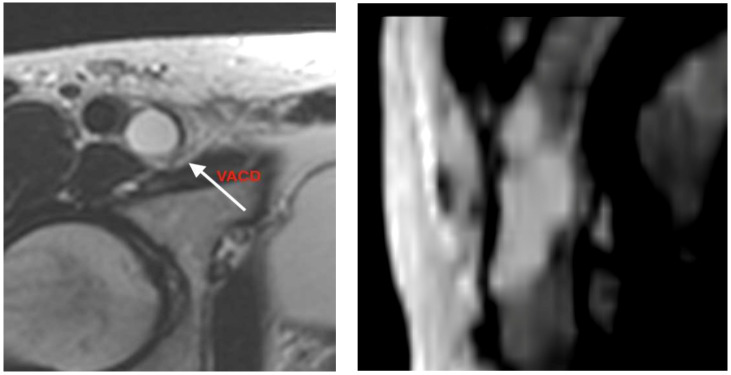
Axial magnetic resonance imaging demonstrating venous adventitial cystic disease, with low signal intensity on T1-weighted images and high signal intensity on T2-weighted images, consistent with mucinous content. The irregular homogeneous structure fills almost the entire lumen. The arrow indicates the cystic irregular lesion.

**Figure 5:**
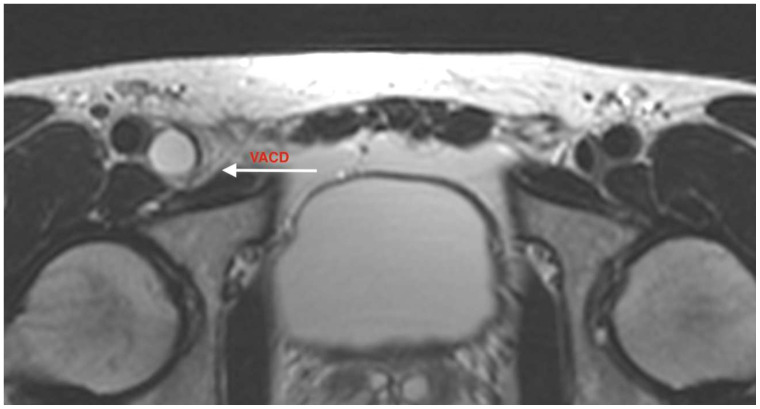
Cross-sectional magnetic resonance imaging demonstrating venous adventitial cystic disease with homogeneous low signal intensity on T1-weighted images and high signal intensity on T2-weighted images, consistent with mucinous content. The irregular homogeneous structure fills almost the entire lumen. The arrow indicates the cystic irregular lesion.

Further imaging with CT angiography (Siemens SOMATOM Edge Plus, Siemens Healthcare GmbH, Erlangen, Germany) and magnetic resonance venography (United Imaging Healthcare uMR 570 1.5T, United Imaging Healthcare Co., Ltd., Shanghai, China) demonstrated a structure localized within the wall of the common femoral vein, consistent with an adventitial cyst occupying approximately 90% of the lumen, without evidence of thrombus (Figure 1, Figure 2, Figure 3, Figure 4 and Figure 5) (OsiriX 14.2.2 MD, Pixmeo SARL, Geneva, Switzerland).

The structure was confined to the common femoral vein, occupied nearly the entire lumen, and was located proximal to the venous bifurcation, which is atypical for thrombus.

## 3. Overview of Treatment and Results

**Figure 6:**
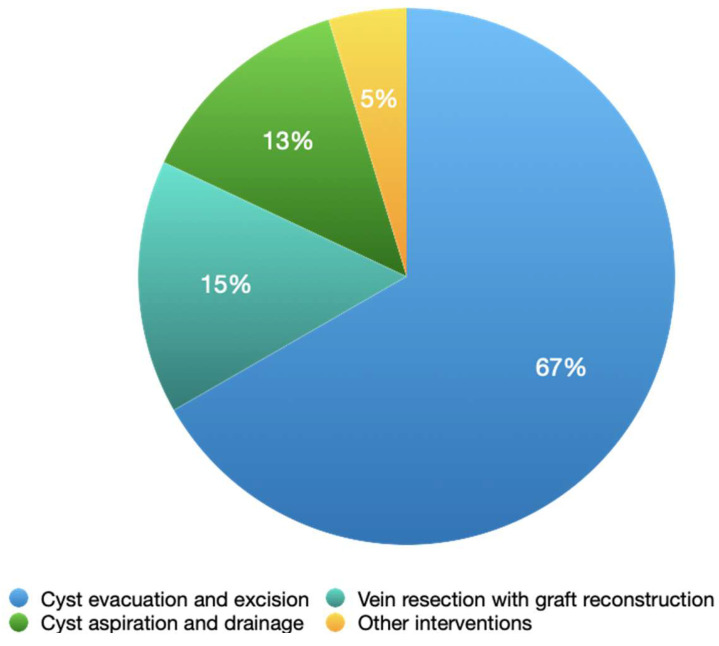
Distribution of treatment approaches for VACD based on pooled data from Bascone 2016; n = 45 [1].

Several treatment options for Venous Adventitial Cystic Disease (VACD) have been described. The most commonly employed approach is cyst evacuation with excision (67%). Less frequently, cyst aspiration and drainage are performed (13%), while vein resection with graft reconstruction accounts for 15% of cases, including the use of autologous vein grafts, other 5% (Figure 6). In cases involving superficial veins, treatment with cyst aspiration and sclerosant injection may be effective in the short term, with recurrence-free periods of up to 18 months.

Surgical intervention is the treatment of choice, given the high risk of recurrence associated with less invasive procedures. Cyst aspiration and drainage are associated with a recurrence rate of up to 83.3%, whereas transadventitial cyst evacuation with cyst excision carries a lower recurrence risk of 7–20% [3]. Overall, the mean recurrence rate of venous adventitial cystic disease (VACD) has been reported to be approximately 26.7%.

In our case, the patient was qualified for open surgical treatment. During the operation, careful dissection revealed a gelatinous cyst within the wall of the common femoral vein, occupying almost the entire venous lumen. After proximal and distal clamping, the cyst was excised together with a portion of the venous wall, followed by patch angioplasty. Histological examination demonstrated a cystic cavity within the adventitia, composed of fibrous tissue containing colloidal mucinous material, separated by thin septal walls. We would recommend standard anticoagulant prophylaxis until surgical intervention, using low molecular weight heparin (LMWH).

The patient was discharged on postoperative day 4, with gradual resolution of symptoms and complete relief of lower-limb swelling.

During a four-year follow-up period, CT angiography was performed annually for the first three years, followed by yearly Doppler ultrasonography, with no evidence of disease recurrence.

## 4. Discussion

Adventitial cystic disease (ACD) is an exceedingly rare vascular disorder, accounting for approximately 0.1% of all vascular diseases [2]. The most common clinical presentation is asymmetric limb swelling [10]. ACD most frequently involves the common femoral artery or vein, with both localizations potentially causing lower-extremity edema originating in the groin region. Many reported cases of ACD have a post-traumatic background, as in the present case; however, the exact pathogenesis remains unclear, and no definitive etiological mechanism has yet been established.

Several theories have been proposed regarding the pathogenesis of VACD, including repetitive microtrauma from mechanical stress or joint movement leading to mucinous degeneration of the venous adventitia [11]. According to the synovial (ganglion) theory, synovial or mucin-secreting cells from adjacent joints migrate along vascular branches or connective tissue planes and subsequently form cysts within the vessel wall [6]. This theory is supported by intraoperative or radiographic visualization of a tract between the cyst and the joint capsule in some cases. Another proposed mechanism, the embryonic theory, explains venous cyst formation as resulting from embryonic rests of mucin-secreting mesenchymal cells trapped within the venous wall during development [5]. Finally, the degenerative theory proposes that local connective tissue degeneration leads to mucin accumulation and subsequent cyst formation [12]. From a clinical perspective, accurate diagnosis remains challenging, as several other conditions may present with similar symptoms.

Sudden unilateral limb edema is a characteristic presentation of deep vein thrombosis, but it may also be caused by less common conditions such as femoral artery aneurysm, ganglion cyst, lipoma, venous leiomyoma [2], malignancy, or lymphadenopathy. Therefore, accurate and thorough differential diagnosis is essential.

## 5. Conclusions

Diagnostic imaging plays a pivotal role in confirming the presence of VACD and differentiating it from other vascular diseases and lesions [1]. Greyscale Doppler ultrasound showing an abnormality localized in the venous wall with cyst morphology. Furthermore, diagnostic modalities, including magnetic resonance imaging (MRI) and CT angiography, used in conjunction with clinical examination and careful comparison with radiologic findings, significantly increase the likelihood of a correct diagnosis. CT venography appears to be superior to MRI and Doppler ultrasonography in this context. Ultrasonography typically demonstrates a single, well-defined structure localized within the common femoral vein, a finding that is more characteristic of adventitial cystic disease. The presence of a solitary, well-circumscribed hypodense lesion arising from the venous wall and enclosed by a capsule on CT venography strongly supports the diagnosis of VACD.

Adventitial cystic disease (ACD) may coexist with or lead to deep vein thrombosis as a result of external venous compression by the cystic mass [3,7]. In such cases, measurement of D-dimer levels may be helpful as an adjunctive diagnostic test. There are no precise data on the number of deep vein thrombosis (DVT) cases caused by venous adventitial cystic disease (VACD). Due to the extreme rarity of VACD, only a few cases of DVT secondary to VACD have been described, accounting for far less than 1% of all DVT cases.

In our opinion, patients with long-standing symptoms such as leg edema, paresthesia, discomfort, swelling, or a palpable mass, in conjunction with a localized hypoechoic lesion on Doppler ultrasonography, should undergo further evaluation with CT and magnetic resonance venography. Multimodal imaging offers the most comprehensive diagnostic information. Doppler ultrasound as a first-line screening examination is a good option because it is a quick, harmless, and patient-accessible test.

This case demonstrates a large lesion occupying nearly the entire lumen of the common femoral vein, with preserved blood flow and no evidence of thrombosis. These features are particularly valuable for diagnostic purposes and for comparison with future computed tomography and magnetic resonance imaging studies. CT and MR angiography also enable detailed assessment of cyst morphology and help exclude communication with adjacent synovial joint capsules, which is essential for surgical planning [13]. Intraoperatively, venous adventitial cystic disease (VACD) presents as a mucinous cyst arising within the vessel wall, affecting either a vein or an artery. Several etiological theories for adventitial cystic disease (ACD) have been proposed, including repetitive microtrauma, ectopic ganglion cell migration, systemic connective tissue disorders, and developmental abnormalities. These mechanisms may involve repeated stretching and distortion of the vessel wall near joints or degeneration of the adventitia, particularly in patients with underlying connective tissue disease. A genetic predisposition may also play a role in the pathogenesis of the disease. Further studies are needed to optimize diagnostic algorithms and refine treatment strategies for this rare condition.

The limited number of reported cases significantly hampers the ability to perform comprehensive scientific investigations. Therefore, further multicenter studies and systematic data collection are needed to improve diagnostic and therapeutic strategies for this rare condition. This report is limited by its single-case design, which restricts the generalizability of the findings. Therefore, the results should be interpreted cautiously and validated in larger, systematic studies.

## Figures and Tables

**Table 1:** Adventitial Cystic Disease localization [3].

ACD (325)
arterial 78% (253)
venous 22% (72)

**Table 2:** Venous Adventitial Cystic Disease localization [3].

Venous ACD Localization	
Common femoral vein	56%
External iliac vein	24%
Popliteal vein.	7%

## Data Availability

The original contributions presented in this study are included in the article. The data presented in this study are available on request from the corresponding author. The data are not publicly available due to privacy restrictions.

## Abbreviations

The following abbreviations are used in this manuscript:

ACD	Adventitial Cystic Disease
DVT	Deep Vein Thrombosis
VACD	Venous Adventitial Cystic Disease
